# The Dynamic Interplay between the Gut Microbiota and Autoimmune Diseases

**DOI:** 10.1155/2019/7546047

**Published:** 2019-10-27

**Authors:** Huihui Xu, Meijie Liu, Jinfeng Cao, Xiaoya Li, Danping Fan, Ya Xia, Xiangchen Lu, Jingtao Li, Dahong Ju, Hongyan Zhao

**Affiliations:** ^1^Beijing Key Laboratory of Research of Chinese Medicine on Prevention and Treatment for Major Diseases, Experimental Research Center, China Academy of Chinese Medical Science, Beijing 100700, China; ^2^Institute of Clinical Medicine, China-Japan Friendship Hospital, Beijing 100029, China; ^3^Graduate School of Peking Union Medical College, Chinese Academy of Medical Sciences/Peking Union Medical College, Beijing 100193, China; ^4^Department of Gastroenterology, China-Japan Friendship Hospital, Beijing 100029, China

## Abstract

The human gut-resident commensal microbiota is a unique ecosystem associated with various bodily functions, especially immunity. Gut microbiota dysbiosis plays a crucial role in autoimmune disease pathogenesis as well as in bowel-related diseases. However, the role of the gut microbiota, which causes or influences systemic immunity in autoimmune diseases, remains elusive. Aryl hydrocarbon receptor, a ligand-activated transcription factor, is a master moderator of host-microbiota interactions because it shapes the immune system and impacts host metabolism. In addition, treatment optimization while minimizing potential adverse effects in autoimmune diseases remains essential, and modulation of the gut microbiota constitutes a potential clinical therapy. Here, we present evidence linking gut microbiota dysbiosis with autoimmune mechanisms involved in disease development to identify future effective approaches based on the gut microbiota for preventing autoimmune diseases.

## 1. The Gut Microbiota

All mammals, including humans, emerge into the world from a sterile environment; thereafter, microorganisms gradually colonize the skin, oral cavity, and nasal, genital, respiratory, and alimentary tract surfaces, which are covered by epithelia [1]. The human gut is colonized by various microorganisms collectively termed the gut microbiota, which has a mutualistic relationship with the host. The gut microbiota is the major source of microbes that may exert beneficial or pathogenic effects on host health. Moreover, the gut microbiota hosted in the gastrointestinal tract, which is the largest host interface exposed to the external environment, comprises approximately two-thirds of the human microbial commensal community [2]. The establishment and development of a beneficial microbiota composition occur during early infancy, influencing health and immune homeostasis in adulthood [3], and disturbing the establishment of this microbiota during early life may have negative effects [4]. Progression of the gut microbiome undergoes the following three phases in early life: the developmental (3-14 months), traditional (15-30 months), and stable (31-46 months) phases. In general, breastfeeding is the most significant factor associated with the development of the microbiome [5].

In addition to the expected role in maintaining gastrointestinal homeostasis, the microbiota is also fundamental for maintaining nutritional activities, metabolic functions in nutrient digestion, detoxification, vitamin synthesis, and immunologic homeostasis in the host. Although the gut microbiota includes viruses, fungi, protozoa, archaea, and bacteria [6], the bacterial component is the most studied and maintains a symbiotic relationship with the host. The bacterial microbiota is divided into aerobic, facultative anaerobic, and obligate anaerobic bacteria according to the degree of aerobic tolerance, with most of the gut microbiota consisting of obligate anaerobic organisms.

The microbiota of the human body consists of more than 10^14^ microorganisms that inhabit different areas of the body, among which the intestine harbors the largest community [7]. The main groups of the gut microbiota in the human intestinal lumen include Firmicutes, Bacteroidetes, Actinobacteria, and Proteobacteria. Due to the expansion of the application of high-throughput deep-sequencing technology in the past decade, it has gradually been revealed that the gut microbiome encodes 3.3 million genes, which is 100-fold more than the number of human genes [8]. Therefore, the gut microbiome is also termed the “human second genome.” Gut microbiota constituents are divided into another three groups according to their functions, called commensal beneficial microorganisms, potentially sensitive pathogens, and pathogenic bacteria. The gut microbiota constituents classified as commensal “beneficial” microorganisms maintain a healthy host environment and offer benefits, also interacting with host tissues in a cooperative and nonpathogenic manner. An imbalance in “sensitive” microorganisms occurs during disease; “pathogenic” microorganisms cause disease, and “therapeutic” microorganisms can help rectify any alterations [9]. The highest species diversity and number are observed in the colon, and various factors affect the composition of the human gut microbiota, including but not limited to diet, age, sex, and geographical location [10, 11]. A change in the microbiota during individual ontogeny is mainly influenced by radical changes in diet, application of antibiotics, or probiotics, and diverse diseases [12].

## 2. The Gut Microbiota and Enteric Mucosal Immunology System

The human mucosa is the site in the human body that most frequently interacts with the complex external environment. The enteric mucosal immunology system relies mainly on gut-associated lymphoid tissue (GALT), which consists of Peyer's patch lymphocytes (PPLs), intestinal intraepithelial lymphocytes (IELs), lamina propria lymphocytes (LPL), and mesenteric lymph nodes (MLNs). IELs include most CD3^+^ T cells, a few B cells, and natural killer (NK) cells; LPLs mainly comprise different subpopulations of T cells and B cells. Our immune system is responsible for the defense against microbial pathogens via recognition and removal. However, another significant role of our immune system is to balance the microbiota inhabiting our mucosal and skin surfaces. The enteric mucosal immune system partially maintains homeostasis by shaping the gut microbial community toward a beneficial effect, and it is essential not only for human health but also for the survival of trillions of microbial community members residing within the intestines. As the gut microbiota in the human body forms a barrier to resist invasion of pathogenic bacteria and synthesis of nutrients, such as proteins and vitamins, changes in the intestinal microbiota impair intestinal mucosal barrier function [13]. With regard to gut barrier homeostasis, the aryl hydrocarbon receptor AhR, which is a ligand-activated transcription factor that recognizes dietary compounds, microbial-derived secondary metabolites, and environmental pollutants to control transcriptional programs in the immune system, plays a key role in the immune response [14]. Because AhR(-/-) mice have insufficient gut barriers, AhR plays a crucial role in sustaining and developing gut barriers. AhR, which is highly expressed in epithelia, is also important for the host gut-microbiome interaction, and AhR expression is significantly decreased in germ-free mice.

The gut microbiota maintains the homeostasis of our immune system. Innate and adaptive immunity plays an important role in the containment and clearance of microbial pathogens. Consequently, subsets of innate and adaptive lymphocytes are considered to sequentially shape the gut microbiota in distinct ways [15]. As a component of one of the most important pathways by which the host recognizes microbial metabolites, AhR participates in the regulation of both the adaptive and innate immune systems, as well as in a variety of diseases and relieving inflammation [16, 17]. Secretory immunoglobulin A (IgA) secreted by the gut mucosa is a major contributor to intestinal mucosal immunity. IgA responses both clear pathogens and promote host-microbial symbiosis, which is driven by the microbiome. For example, *Bacteroides fragilis* regulates its surface to encourage binding of IgA in vivo, which facilitates bacterial adherence [18]. The repertoire of IgA bound to the gut microbiota is related to both T-cell-dependent and T-cell-independent pathways. AhR signaling can modulate the dynamics of the gut microbiota, which might be involved in the interaction between host metabolism and the gut microbiota [19]. Furthermore, activation or disruption of AhR can influence the microbiota [20], and specific dietary and microbial metabolites of tryptophan can serve as the ligand for AhR [21]. After tryptophan metabolite binding and activation of AhR, the transcription factor induces expression of downstream cytokines, including IL-22 and IL-17, to modulate intestinal homeostasis [22]. Ozcam et al. used whole-genome sequencing, comparative genomics, and targeted gene inactivation to show that *Lactobacillus reuteri* R2lc and 2010 harbor an orthologous polyketide synthase (PKS) gene cluster that serves as a novel pathway responsible for AhR activation [23].

Gut dysbiosis not only affects the expression level of Toll-like receptors (TLRs) of antigen-presenting cells but also contributes to Th17/Treg imbalance [24]. The composition and metabolites of the gut microbiota have major roles in producing antibodies, shaping the B cell repertoire, maintaining the Th17/Treg balance, regulating different subpopulations of Th17 cells, and regulating homeostasis in different populations of T helper cells [25]. The promotion of gut regulatory T cells via AhR signaling is important for gut immune homeostasis. In a T cell-transfer genetic mouse model of colitis, AhR-expressing Tregs exhibited increased suppressive activity compared with Tregs lacking AhR expression [26]. AhR regulates immune surveillance of the gut by retaining IELs and redistributing the Th17/Treg cell balance. Studies have also shown that AhR expression plays a fundamental role in contributing to the establishment of the intestinal microbial community structure in mice [27].

In collagen-induced arthritis (CIA) rats, the intestinal mucosal immune response is enhanced and immune tolerance is disturbed. Moreover, madecassoside treatment can increase relative expression of forkhead box P3 (FoxP3) mRNA in the small intestine, downregulate concentrations of sIgA and IFN-*γ* in the small intestinal content and tissue, respectively, decrease the ratio of CD4^+^/CD8^+^ cells in the epithelium and laminar propria, and decrease relative expression of CD80, CD86, IL-6, and IL-12 mRNA in CIA rats to downregulate the intestinal mucosal immune response and restore normal immune tolerance [28]. Partial immunosuppressive activities of triptolide that lower arthritis scores and upregulate the TGF-*β* level might occur through an effect on enteric mucosal immune lymphocytes in Peyer's patches, IELs, and LPLs of DBA/1 with CIA [29]. Additionally, a Gui Zhi decoction was found to lower the number of slgA+ cells and CD4^+^ and CD8^+^ T cells in the lamina propria in CIA mice and downregulate IL-6 and TNF-*α* concentrations in serum, revealing that the mechanism of decreased incidence of CIA occurs in part through the regulation of enteric mucosal immunity in mice [30].

## 3. The Gut Microbiota and Host Health

Manipulation of the dense gut microbiota has broad implications for host health. The diverse human gut microbiota plays a fundamental role in the well-being of hosts and is closely correlated with the growth, development, substance metabolism, and immune function of the host [31]. Indeed, the gut microbiota is intimately connected to numerous facets of host biology, and beneficial microbiota organisms play an important role in the processes of food digestion, immune system homeostasis maintenance, anti-infection immunity support, lipid metabolism modulation, and others [32]. Specifically, the gut microbiota can influence intestinal functional integrity, barrier strength, and permeability regulation, thereby influencing the immune response, which has been linked to the development of inflammatory disease. Studies have shown that high levels of *Prevotella copri* correlate with low levels of the gut microbiota previously associated with immune-regulating properties [33]. Nutrient availability may also influence the gut microbiota and expand the possibility of cell-based therapeutic strategies in the gut. For example, Shepherd et al. demonstrated that the transfer of the porphyran utilization locus into an exogenous *Bacteroides* strain in the mouse gut can control strain abundance by multiple orders of magnitude [34]. Differences between microbiota profiling of AhR(+/+) and AhR(-/-) mice fed a diet enriched with a specific AhR ligand or a diet depleted of any known AhR ligands have been reported; thus, the AhR signaling pathway may influence microbiome composition in the small intestine. Microbiome metabolites, such as short-chain fatty acids (SCFAs), also regulate AhR and its target genes in the intestine [35].

An imbalance of the gut microbiota might underlie a broad spectrum of human illnesses, such as immune, metabolic, and cardiovascular diseases as well as neurological disorders, and alterations of the gut microbiome may be carried out in the future to improve clinical outcomes across diverse genetic backgrounds. The gut microbiome may also improve the prediction accuracy of human traits, including glucose measures and obesity [36]. Host genetic factors play a minor role in determining the microbiome composition compared with factors associated with drugs, diet, and anthropometric measurements [37]. Based on epidemiological data, the propensity for horizontal transmission of bacterial genera representing pathogenic genera in humans may be responsible for hospitalizations [38]. AhR is a pleiotropic factor that might influence the community structure of the gut microbiota. By utilizing C57BL6/J AhR(-/+) and AhR(-/-) cohoused littermates after 18 days of genotypic segregation, Murray et al. found significant changes in the phylum abundance of the gut microbiota, particularly for *Verrucomicrobia* and segmented filamentous bacteria (SFB). Transfer of the AhR(-/-) microbiota to wildtype germ-free mice indicated Ahr(-/-)-microbial dependence, which increased *Verrucomicrobia* abundance and inflammatory tone [27]. AhR is also a potential target in the treatment of antibiotic-associated gut barrier damage. Wang et al. showed the downregulation of AhR, IL-22, and phosphorylated Stat3 (p-Stat3) with decreasing antimicrobials but that antimicrobial molecule levels were significantly rescued after antibiotic treatment when given the exogenous AhR agonist 6-formylindolo (3,2-b) carbazole (Ficz) [39]. AhR is a promising therapeutic target in immune-mediated diseases because it can induce IL-22 and regulate anti-inflammatory signals as well as control the differentiation, proliferation, and activation of immune cells.

## 4. The Gut Microbiota and Autoimmune Diseases

Autoimmune diseases are characterized by aberrant production of autoantibodies. Genetic and/or environmental factors act on the immune system and cause abnormal generation of autoantibody-producing B cells and autoreactive T cells and anomalous production of proinflammatory cytokines. It has been hypothesized that the increasing incidence of autoimmune diseases is due to considerable shifts in the gut microbiota among multifactorial reasons following dietary changes and the widespread application of antibiotics (Table 1).

Both genetic and environmental factors contribute to autoimmune diseases, including complex genetic elements, geographical location, patient exposure, immunologic derangement, and viral infections. By binding diverse dietary, cellular, and microbe-derived ligands, AhR might be involved in autoimmune diseases via the transduction of extrinsic and intrinsic signals into cellular responses [40]. According to Qiu et al., reduced innate IL-22 levels in AhR-deficient mice allowed the expansion of commensal SFB (an immune activator), promoting Th17 cell proliferation. Innate expression of AhR plays a protective role in T-cell-mediated experimental colitis by suppressing pathogenic Th17 cells [41]. In addition, Stedtfeld et al. reported that compared with levels under a traditional gut microbiome, 2,3,7,8-tetrachloro dibenzo-*p*-dioxin (TCDD, an AhR ligand) induced shifts in the abundances of SFB and *Bacteroides fragilis* (an immune suppressor) in mice. Moreover, the TCDD-induced host response was significantly regulated by the presence of SFB in the gut microbiome, demonstrating therapeutic potential between AhR ligands and key commensals [42]. Dysbiosis of the gut microbiota has been identified as a potential factor that causes autoimmune diseases, which in humans are attributed to multiple factors, even though the relative contribution of the gut microbiota remains elusive. Posttranslational modification of autoantigens and cross-reactivity with autoantigens represent mechanisms by which the gut microbiota mediates autoimmunity at the molecular level. At the cellular level, translocation of live gut bacteria across a dysfunctional gut barrier provokes direct interactions with immune and tissue cells and subsequently instigates systemic autoimmunity [43].

The association between gut microbiota imbalance and autoimmune diseases may be due to several mechanisms that may impact the human immune system and function. For instance, modulating the host immune response and activation of antigen-presenting cells (APCs), including dendritic cells (DCs), may evoke antigen presentation and cytokine production, subsequently affecting T cell differentiation and function. Furthermore, this impact disrupts the homeostasis between T helper 17 (Th17) cells and T regulatory cells (Tregs). There may also be similarities between foreign antigens and self-antigens attributed to antigenic mimicry; consequently, pathogen-derived autoreactive T and B cells are activated, thus promoting autoimmunity. However, the permeability of the intestinal mucosa is altered because expression of tight junction (TJ) proteins is modulated.

Abundant evidence suggests that the gut microbiota may be involved in the initiation and amplification of disease progression in patients with autoimmune diseases. The possible mechanisms include molecular mimicry, impacts on intestinal mucosa permeability, the host immune response caused by the microbiota, and antigenic mimicry. Alterations in gastrointestinal microbial communities have been linked to autoimmune diseases. The gut microbiota can influence or interfere with immune sensing in discriminating between self and nonself, which may contribute to autoimmune diseases. Patients with autoimmune diseases commonly display signs of impaired gut barriers, which may result in immune exposure to commensal gut bacteria. In addition, a breakdown in mucosal immune tolerance leads to aberrant and pathological immune responses toward the gut microbiota, which contributes to disease severity.

In recent years, an increasing number of studies have shown that several approaches, including antibiotics, prebiotics, antimicrobial interventions, fecal microbiota transplantation (FMT) [44], and selective probiotics, can regulate the gut microbiota [45, 46]. However, some inappropriate antibiotic use and even nonantibiotic (human-targeted) drugs have been correlated with changes in gut microbiota composition. Growing experimental and clinical evidence has suggested that the chronic inflammatory response induced by gut microbiota dysbiosis can strongly contribute to the development of autoimmune diseases. Overall, germ-free animal models are more suitable for exploring the influence of the host microbiome on the course and development of many disorders [47, 48]. In general, the microbiota may trigger autoimmunity in genetically susceptible individuals or prevent autoimmunity in others.

### 4.1. The Gut Microbiota and Rheumatoid Arthritis

Rheumatoid arthritis (RA) is a systemic, inflammatory, and autoimmune disease characterized by the destruction of cartilage and bone, ultimately progressing to functional disability. RA is one of the most common autoimmune diseases, affecting approximately 1% of the worldwide population and occurring twice as frequently in women than in men. It has been demonstrated that RA correlates with the inflammatory response mediated by CD4^+^ Th1 and Th17 lymphocytes and the imbalance between Th17 lymphocytes and Tregs, and the complex interaction between genes and environmental factors is implicated in the etiology and progression of RA. Talbot et al. demonstrated that cigarette smoking induces experimental arthritis aggravation and increases the frequency of Th17 cells in vivo, and experiments in vitro have also shown that cigarette smoking directly promotes Th17 differentiation. Furthermore, the effects of cigarette smoking on inducing arthritis aggravation are AhR dependent, and environmental pollutants with AhR agonist activity exacerbate arthritis by directly enhancing Th17 cell differentiation [49]. Nevertheless, berberine (BBR) promotes the differentiation of CD4^+^ CD25^+^ Treg cells by inducing FoxP3 activation via AhR to regulate the Th17/Treg imbalance in RA [50]. The emerging field of microbiota research has raised awareness that the gut microbiota and its metabolites interact with the host immune system, with associations with the pathogenesis of RA. Moreover, changes in the gut microbiota have been implicated in the loss of tolerance of self-antigens and in increasing inflammatory events that promote joint damage.

The most commonly used experimental animal model is CIA. A recent experimental study revealed that the gut microbiome can influence arthritis susceptibility. Differences in the gut microbiota were consistently detected between CIA-susceptible and CIA-resistant or healthy mice. Before arthritis onset, the susceptible mice showed enriched operational taxonomic units (OTUs) related to *Lactobacillus* as the dominant genus. As arthritis developed, the abundances of families Bacteroidaceae, Lachnospiraceae, and S24-7 increased significantly in CIA-susceptible mice. When transplanted into germ-free mice, the microbiota showed a higher frequency of CIA induction in animals receiving the CIA-susceptible microbiota than in those receiving the microbiota from CIA-resistant mice [47]. In addition, Sato et al. found that CIA was aggravated in mice by oral administration of *Porphyromonas gingivalis*, significantly altering the gut microbiome, with concomitant elevation in interleukin- (IL-) 17 levels in sera, gut barrier function impairment, and increasing Th17 cell proportions among mesenteric lymphocytes [51]. Liu et al. investigated the effect of *Lactobacillus salivarius* isolated from RA patients on CIA mice by oral administration for 5 weeks starting at 2 weeks before arthritis induction, and the bacterium exhibited antiarthritic and anti-inflammatory effects. Furthermore, pretreatment with *L. salivarius* induced profound changes in the cellular adaptive immune response by reducing and increasing the Th17 and Treg cell fractions, respectively [52].

RA patients exhibit decreased gut microbiota diversity compared with controls, which correlates with RA duration and autoantibody levels. Studies of the gut microbiota have shown that RA is characterized by an increase and/or decrease in the abundance of microbe groups compared with that in healthy individuals. A previous taxon-level analysis suggested that *Actinobacteria* levels are decreased among abundant taxa in RA patients compared with that in controls. Based on a random forest algorithm, it has been suggested that *Collinsella*, *Eggerthella*, and *Faecalibacterium* are related to RA. The abundance of *Collinsella* was associated with increasing levels of alpha-aminoadipic acid and asparagine as well as production of IL-17A, and the role of *Collinsella* in altering gut permeability and RA severity in experimental arthritis has been confirmed [53]. The composition of the gut microbiota in RA patients free of therapy is characterized by certain abnormalities compared to that in healthy controls [54]. A data-driven study suggested that human gut microbial metabolites directly interact with 18.1% of all 166 RA-associated genes, participate in 71.4% of 311 RA-associated genetic pathways, and affect 51.3% ofRA-related phenotypes, including immune system pathways and phenotypes; this in turn demonstrated that the gut microbiota and its metabolites contribute to RA at genetic, functional, and phenotypic levels [55]. Another study reported that the HLA-B27 and HLA-DRB1 alleles affect susceptibility to RA, with effects on the gut microbiome that partially cause or increase the risk of RA [56]. In addition, changes in fecal bacteria may represent the RA condition or the outcome of RA progression. Additional significantly affected bacterial species and increased bacterial diversity and abundance have been observed in patients with early RA based on denaturing gradient gel electrophoresis (DGGE); the fecal microbiota (FM) of patients with early RA contains significantly more *Lactobacillus* (*L. salivarius*, *L. iners*, and *L. ruminis* becoming the predominant bacteria) than a healthy control group, and the presence of *L. mucosae* was also observed [57]. Regardless, the same gut microbiota species might not play a concurrent role in the pathogenesis of RA, and different species may produce different effects on RA. For instance, the abundance of *Prevotella copri* is increased in some patients with early-stage RA, but *Prevotella histicola* from the human gut microbiota suppresses the development of RA [58]. Interestingly, Hablot et al. evaluated the impact of experimental colitis on the development of CIA in mice and observed that concomitant experimental colitis reduced severity in association with modification of the gut microbiota composition [59].

Once altered, the gut microbiota can be partially restored through disease-modifying antirheumatic drugs. Restoration-altered gut microbial ecosystems may have therapeutic effects, and this interaction has an impact on clinical outcome in RA. Xiao et al. have suggested that *Paederia scandens* extract has a protective effect on CIA mice by decreasing the relative abundance of inflammatory-related microorganisms, including *Desulfovibrio*, *Helicobacter*, *Mucispirillum*, and Lachnospiraceae [60]. Gut dysbiosis is a hallmark of RA with different serological and clinical parameters. The phylum Euryarchaeota correlates directly with the disease activity score on 28 joints (DAS-28), emerging as an independent risk factor. RA patients under treatment with immunosuppressive drugs, such as etanercept, present partial restoration of a beneficial microbiota [54]. Although RA patients may require antibiotic treatment, partial depletion of the natural gut microbiota by oral Baytril (enrofloxacin) aggravates arthritis symptom severity in CIA mice [32]. Therefore, precautions should be taken when RA patients are treated with drugs, as some of them may aggravate the disease.

### 4.2. The Gut Microbiota and Systemic Lupus Erythematosus

Systemic lupus erythematosus (SLE) is a complex, chronic, and inflammatory prototypical autoimmune disease characterized by persistent inflammation in multiple organs of the body. SLE most often affects women but is triggered by complicated interactions of different factors (genetic predisposition, hormonal alteration, and environmental factors, among others) that are not completely clear at present. Alterations in the composition of the microbiota lining the intestines are suspected to be involved in the etiopathogenesis of SLE, and a number of studies have been carried out to demonstrate that gut microbiota dysbiosis affects the onset and development of SLE. Specified genera of the microbiota can be used to distinguish SLE patients from healthy individuals. A recent study revealed a decrease in Ruminococcaceae and an increase in Proteobacteria in patients with SLE in northeastern China [61]. In addition, compared with control group subjects, SLE patients were characterized by a decrease in bacterial richness, a lower Firmicutes/Bacteroidetes ratio, and an increase in the relative abundance of *Bacteroides* species in fecal samples. In studies of human SLE and murine models, gut microbiota compositions have been shown to differ from those of healthy controls. Zegarra-Ruiz et al. found that *Lactobacillus* in feces is enriched in a subset of SLE patients, and using Toll-like receptor 7- (TLR7-) dependent mouse models of SLE, *Lactobacillus reuteri* was shown to exacerbate autoimmune manifestations, which was inhibited by dietary resistant starch (RS) via SCFAs [62]. Ligands of AhR involved in the pathogenesis and development of SLE have also been identified [63]. Previous data from a hospital-based case-controlled study showed that the expression level of AhR in PBMCs was significantly higher in SLE patients than in healthy controls and that the proportion of AhR-overexpressing cells to Th17 cells was significantly increased in SLE patients compared with that in the control group. In the high-AhR ratio group, there were more younger SLE patients with skin lesions and ultraviolet allergies and lower complement 3 levels than in the low-AhR ratio group, indicating AhR as a potential biomarker for predicting SLE skin injury [64]. Mohammadi et al. reported that the AhR agonist indole-3-carbinol (I3C) mediates AhR activation, contributing to immunoregulatory effects on macrophages of SLE patients by decreasing expression of proinflammatory cytokines and overexpression of anti-inflammatory cytokines [65].

Obvious dysbiosis of the gut microbiota is associated with active and remissive SLE patients and thus may be used to diagnose SLE and predict disease activity. SLE patients reportedly exhibit characteristic patterns of gut microbiota dysbiosis that directly parallel disease activity. Among SLE-related microbiota, the genera *Streptococcus*, *Campylobacter*, and *Veillonella* and species *S. anginosus* and *E. dispar* were found to be positively associated with lupus activity; in contrast, the genus *Bifidobacterium* correlated negatively with disease activity. Phylogenetic Investigation of Communities by Reconstruction of Unobserved States (PICRUSt) analysis showed that metabolic pathways are different not only between SLE patients and healthy controls but also between active and remissive SLE patients [66]. Fecal 16S rRNA analyses were performed in a cross-sectional discovery cohort, showing that patients with SLE had lower species richness diversity than did controls, with obvious reductions in taxonomic complexity in patients with a high SLE disease activity index. It was also found that SLE patients had an overall 5-fold increase in the representation of *Ruminococcus gnavus* (Lachnospiraceae) and that intestinal expansions of *R. gnavus* were directly proportional to overall disease activity and most pronounced in patients with lupus nephritis [67]. SLE patients with active lupus disease possess an altered gut microbiota that differed in the genera *Odoribacter* and *Blautia* as well as an unnamed genus in the family Rikenellaceae and exhibit increased representation of gram-negative bacteria. Gut microbiota dysbiosis has also been reported in experimental murine lupus models, but differences between murine models and human subjects were found, including with regard to diversity. Nonetheless, the composition of the gut microbiota changed significantly from the predisease stage to the lupus disease stage in NZB/W F1 mice, with increased diversity and representation of some bacterial species. Using dexamethasone as an intervention to treat NZB/W F1 mice, an increased abundance of a group of lactobacilli in the gut microbiota might be correlated with enhanced disease severity [68].

Free fatty acids (FFAs) are upregulated in the serum of SLE patients, and the production of SCFAs plays an important role in the metabolism modulated by the gut microbiota. As an example, Rodriguez-Carrio et al. investigated potential links among total and specific serum FFA levels, fecal SCFA levels, and gut microbiota composition, and the findings revealed that altered gut microbiota composition in SLE patients is linked to altered SCFA production and increased FFA levels in serum [69]. Oxidative stress has been implicated in SLE, with the requirement of a balance in the gut microbiota and oxidant-antioxidant ratios. Gonzalez et al. compared levels of blood antioxidants and the gut microbiota with serum malondialdehyde (MDA) and C reactive protein (CRP) levels in 21 subjects with nonactive SLE; in this study, the serum copper content was positively associated with CRP levels, and CRP was also positively associated with the proportion of *Lentisphaerae*, Proteobacteria, and Verrucomicrobia in feces. In addition, MDA levels displayed negative associations with the proportions of *Cyanobacteria* and Firmicutes, though the proportion of *Actinobacteria* showed a positive correlation [70]. In general, the gut microbiome profiles of SLE patients are more influenced by disease than by ethnicity, and SLE patients from both China and Spain show depletion of Firmicutes and enrichment of *Bacteroidetes*. Additionally, numerous gut microbiota genera were identified as SLE-related microorganisms in Chinese subjects, with significant enrichment of the genera *Rhodococcus*, *Eggerthella*, *Klebsiella*, *Prevotella*, *Eubacterium*, *Flavonifractor*, and *Incertae sedis* and significant depletion of the genera *Dialister* and *Pseudobutyrivibrio* [71].

SLE is correlated with increased intestinal permeability that results in increased systemic microbial exposure. Reducing microbial exposure or improving barrier function may provide therapeutic approaches for SLE patients [72]. In fact, targeting the gut microbiota with drugs may be one of the mechanisms for treating SLE. For instance, treatment with a conjugate of tuftsin and phosphorylcholine (termed TPC) improved both immune and disease parameters of SLE, which was accompanied by a reduced abundance of Akkermansia and correlated with the clinical and serological parameters of lupus and an increased abundance of *Bifidobacterium*; Turicibacter; unclassified Mogibacteriaceae; unclassified *Clostridiaceae*; *Adlercreutzia*; *Allobaculum*; and Anaeroplasma [73]. Oral antibiotic administration during active SLE disease might reshape the gut microbiota, remove harmful Lachnospiraceae, increase the relative abundance of *Lactobacillus* spp., attenuate SLE-like disease in lupus-prone mice termed MRL/lpr mice, and ameliorate systemic autoimmunity and kidney histopathology when given after disease onset [47]. A study with in vitro cultures revealed that *Bifidobacterium bifidum* supplementation prevented CD4^+^ lymphocyte overactivation, supporting a possible therapeutic method of probiotics containing Treg-inducer strains to restore the Treg/Th17/Th1 imbalance in SLE [74]. Because previous research demonstrated that the beneficial anti-inflammatory effects of *Lactobacillus* treatment were observed in female and castrated male mice but not in intact males, the gut microbiota may control lupus nephritis in a sex hormone-dependent manner [75].

### 4.3. The Gut Microbiota and Spondyloarthritis

Spondyloarthritis (SpA) comprises a group of several related diseases with distinct phenotypes, including ankylosing spondylitis (AS), reactive arthritis (ReA), psoriatic arthritis (PsA), arthritis related to inflammatory bowel disease (IBD), and a subgroup of juvenile idiopathic arthritis. Both environmental and genetic factors are responsible for the onset and development of SpA; however, the cause of SpA remains to be elucidated. The presence of HLA-B27 has a direct effect on SpA development [76]. Among nongenetic factors, it has been observed that the gut microbiota is distinct in early SpA subjects compared with that in controls, and an unbalanced gut microbiota possibly mediates activation of the inflammatory pathways observed in SpA. Previous studies have revealed that the proportions of certain gut bacterial families were increased in SpA patients, including Bacteroidaceae, Porphyromonadaceae, and Prevotellaceae [77]. Gill et al. demonstrated that the effects of HLA-B27 on the gut microbiota and dysbiosis in SpA are strongly associated with the host genetic background and/or environment, despite convergence of dysregulated immune pathways. Histologic analysis showed that both HLA-B27-transgenic Lewis rats and HLA-B27-transgenic Fischer rats developed gut inflammation but that DA rats were resistant to the effects of HLA-B27; HLA-B7-transgenic rats of the control group were not affected. Gut microbial changes in HLA-B27-transgenic rats were strikingly divergent among the 3 different host genetic backgrounds, including different patterns of dysbiosis in HLA-B27-transgenic Lewis and HLA-B27-transgenic Fischer rat strains, with some overlap. The resistance of DA rats to SpA might be due to the lack of SFB, which promotes CD4^+^ Th17 cell development [78].

Colonization of particular intestinal bacteria was sufficient to induce a disparate phenotypical SpA disorder. The microbiota of AS patients was characterized by an increased abundance of Proteobacteria based on 16S rRNA gene- and ITS2-based DNA sequencing, possibly due to the enrichment of *Escherichia*-*Shigella*, *Veillonella*, and *Lachnospiraceae* NK4A136 group, and was also characterized by a reduction in *Prevotella* strain 9, *Megamonas*, and *Fusobacterium*. Moreover, the gut microbiota of AS patients features increasing levels of Ascomycota, especially of the class Dothideomycetes, and a decreasing abundance of Basidiomycota, mainly owing to a reduction in Agaricales. Moreover, decreased ITS2/16S biodiversity ratios and altered bacterial-fungal interkingdom networks were observed in AS patients compared with those in healthy controls [79]. Additionally, significant increases in the abundance of *Erwinia* and *Pseudomonas* and an increased prevalence of typical enteropathogens associated with ReA were found in ReA patients. Subjects with ultrasound evidence of enthesitis display an enrichment of *Campylobacter*, but subjects with uveitis and radiographic sacroiliitis are enriched in *Erwinia* and *unclassified Ruminococcaceae*, with both having *Dialister* enrichment [80].

Gut inflammation is closely linked to chronic joint inflammation. DNA from several mucosal bacteria, such as *Prevotella* spp., can be found in other tissues in addition to the intestine of the host, including the spleen, liver, lung, serum, MLN, eyes, and ankle joints [81]. More than half of SpA patients experience microscopic gut inflammation, often resembling early Crohn's disease (CD) [82], and there is a significant difference in the intestinal microbial composition in SpA patients who have microscopic gut inflammation compared to those without microscopic signs. The abundance of the genus *Dialister* was found to be positively correlated with the AS disease activity score, and a reduced frequency of *Dialister* was further observed in noninflamed ileal and colonic biopsy tissues from SpA patients and healthy controls [83]. Breban et al. performed 16S ribosomal RNA gene sequencing on fecal DNA isolated from stool samples in two consecutive cross-sectional cohorts comprising three groups of SpA and RA patients and healthy controls. Compared with both RA patients and healthy controls, SpA patients exhibited a significant increase in the abundance of RG, which may act as a marker of disease activity in patients who have a history of IBD [84]. Viladomiu et al. also evaluated the fecal microbiome of IBD patients with or without peripheral SpA, revealing selective enrichment in IgA-coated *Escherichia coli* in those with Crohn's disease-associated SpA (CD-SpA) compared to that in those with CD alone. Colonization of germ-free mice with CD-SpA *E. coli* isolates induces Th17 mucosal immunity. Furthermore, when modeling the increase in mucosal and systemic Th17 immunity, colonization of IL-10-deficient or K/BxN mice with CD-SpA-derived *E. coli* led to more severe colitis or inflammatory arthritis, respectively [85].

The microbiota may be utilized as a biomarker in the future to predict clinical response in SpA. An exploratory study investigated an increased proportion of the Burkholderiales order in future responder patients with SpA before three months of anti-TNF-*α* treatment; modifications in microbiota composition were observed after treatment, but modification in a specific taxon was not found, regardless of the clinical response [86]. Microbiota-dependent intestinal inflammation drives the systemic inflammatory and osteoclastogenic potential of the monocyte compartment in the HLA-B27-transgenic (B27-Tg) rat model of SpA [87]. Treating HLA-B27/*β*2-macroglobulin- (*β*2m-) transgenic rats, which are a model of SpA, with the SCFA propionate for 10 weeks attenuated the development of HLA-B27-associated inflammatory disease, and this result may provide a novel therapy of microbial metabolites for HLA-B27-associated SpA [88].

### 4.4. The Gut Microbiota and IBDs

IBDs are subdivided into CD and ulcerative colitis (UC), which are chronic inflammatory diseases of the gastrointestinal tract resulting from an inappropriate immune response. Etiological factors of IBD may include personal genetic susceptibility, immune responses, the intestinal microbiota, and environmental stimuli. IBD is one of the most common diseases associated with gut microbiota dysbiosis, and colonization by a particular intestinal bacterium is sufficient to induce the onset of IBD as well as disease exacerbation. A recent study suggested that alteration of intestinal extracellular vesicle proteins might mediate the aberrant host-microbiota interactions in pediatric IBD [89]. IBD patients exhibit hallmarks of stool microbiome dysbiosis, with loss of a diversified core microbiome, depletion of specific beneficial bacteria, and/or enrichment of bacterial virulence factors compared with those in healthy controls [90]. Omori et al. analyzed fecal samples by 16S rRNA gene next-generation sequencing and revealed that the proportions of the family Paraprevotellaceae and the genus *Porphyromonas* were significantly increased in IBD dogs compared to those in healthy dogs [91]. IELs are T cells that most closely contact intestinal bacteria and may be influenced by the dissimilarity in microbiota constituents in distinct subtypes of IBD. A previous study indicated that IELs and IEL-produced cytokines correlated positively and negatively with the relative abundance of multiple bacterial taxa. Compared to those in healthy controls, IELs from subjects with UC secreted significantly greater amounts of IL-1*β*, and those from subjects with CD secreted significantly higher amounts of IL-17A, IFN-*γ*, and TNF-*α* [92]. In general, interaction between the host genome and the gut microbiota based on individual differences might influence health. Healthy individuals with a high genetic risk for IBD display a decrease in the genus *Roseburia*. Additionally, the site of disease is a major determinant of the gut microbiota, with patients with ileal CD exhibiting a decrease in *α* diversity compared to those with colonic CD [93].

Gut microbiota signatures have potential in IBD prognosis and diagnosis as well as in evaluating disease activity for IBD. Zhou et al. revealed by meta-analyses that gut microbial alteration patterns in IBD were similar among Chinese and Western populations. However, these authors found relatively increased levels of *Actinobacteria* and *Proteobacteria* (Enterobacteriaceae) and a relatively decreased level of *Firmicutes* (Clostridiales), which were strongly related to IBD severity [94]. Altomare et al. have suggested that the gut mucosal-associated microbiota (MM) better discriminates IBD differential patterns from those of healthy controls than does the FM. They found that FM profiles in IBD patients were more similar to those in healthy controls than were MM profiles. Some microbiota are apparently altered in the MM of IBD patients, showing a statistically significant increase in Proteobacteria and a decrease in Firmicutes and Actinobacteria proportions compared to those of healthy controls [95]. *Clostridium difficile* infection (CDI), which is a common complication in IBD, may also cause poor IBD outcomes. Sokol et al. performed a study using 16S sequencing and analysis by the QIIME pipeline and found that IBD patients with CDI had more specific microbiota dysbiosis with higher levels of RG and Enterococcus OTUs and lower levels of *Blautia* and *Dorea* OTUs than did IBD patients without CDI [96]. Additionally, differences in the gut microbiota might distinguish patients with IBD from those with other intestinal-related diseases. By combining species-level profiles and strain-level profiles with bacterial growth rates, metabolic functions, antibiotic resistance, and virulence factor analyses, patients with irritable bowel syndrome (IBS) showed a distinct microbiota composition compared with that of IBD patients [97]. Changes in the microbiota and microbiota-stool bile acid also allow for good separation of patients with primary sclerosing cholangitis-associated inflammatory bowel disease (PSC-IBD) and those with IBD alone, and this situation might predispose patients to future colorectal neoplasia [98]. Both the activity and presence of the gut microbiota play an important role in IBD. Schirmer et al. detected organisms, such as *Dialister invisus*, that were metagenomically abundant but inactive or dormant in the gut with little or no expression. Certain disease-specific microbial characteristics were more pronounced or detectable at only the transcript level, such as pathways predominantly expressed by different organisms, in a host with IBD [99].

IBD patients exhibit lower diversity and richness of gut microbiota; thus, restoration of certain gut microbiota diversity is beneficial for treating and predicting clinical effectiveness in IBD patients. A significant increase in the relative abundance of Clostridiales was observed in response to infliximab treatment compared to that during relapse. Moreover, the relative abundance of Clostridiales was able to predict treatment effectiveness with 86.5% accuracy alone and 93.8% accuracy in combination with calprotectin levels and a CD activity index [94]. Kalenyak et al. evaluated the intestinal microbiota of dogs with IBD and showed that an unclassified genus of Neisseriaceae was abundant in the duodenum but that after treatment, *Bacteroides* reached significant abundance in the colon [100]. FMT is an emerging therapy and may be a potential treatment approach for IBD. Knoll et al. assessed the composition of the fecal microbiome by comparing pediatric CD and UC patients to their healthy siblings. Species richness and diversity were significantly reduced in UC children, with significant reductions in the abundance of *Eubacterium rectale* and *Faecalibacterium prausnitzii*. *Escherichia coli*, the abundance of which correlates positively with certain virulence genes, was enriched in UC children. Microbiota remodeling therapy from family donors will be a viable option for UC children [101]. Fasting-mimicking diet (FMD) cycles are also a potential therapy to ameliorate IBD, with a protective effect on the gut microbiota composition in DSS-induced mice [102].

By promoting clinical and endoscopic benefits, AhR expression may be a therapeutic target of interventions for IBD. AhR agonists are among the immunomodulators that can maintain immune tolerance. Lyer et al. identified that dietary- and microbial-derived oxazoles induced CD 1d-dependent intestinal inflammation via the action of AhR in the intestinal epithelium [103]. Caspase recruitment domain family member 9 (CARD9) is a susceptibility gene for IBD, and Card9 (-/-) mice are more susceptible than are wildtype mice to colitis. Intestinal inflammation of Card9 (-/-) mice was attenuated after inoculation with three *Lactobacillus* strains capable of metabolizing tryptophan or when treated with an AhR agonist [104]. Aoki et al. suggested that indole-3-pyruvic acid (IPA), which is a major precursor of microbiota-derived AhR agonists, potentially improves the severity of experimental chronic colitis in a mouse model by regulating the frequency of different T cells [105]. In dextran sodium sulfate- (DSS-) induced colitis, a deficiency in AhR in mouse intestinal epithelial cells exacerbates inflammation [106]. Marafini et al. showed the benefit of AhR-dependent regulatory effects in an experimental mouse model of colitis by testing the modulatory effect of the compounds NPD-0414-2 and NPD-0414-24, which are chemical ligands of AhR that can induce additional IL-22. Mice given NPD-0414-2 and NPD-0414-24 developed a significantly less severe form of TNBS colitis following decreased expression of IFN-*γ* and increased expression of IL-22. Moreover, the therapeutic effect of these two derivatives on ongoing colitis was abrogated in AhR-deficient mice [107]. Oh-Oka et al. found that mesalamine, a first-line drug for treating IBD, exhibited an anti-inflammatory effect by inducing Tregs in an AhR-dependent manner and an increased level of the active form of TGF-*β* [108].

It is worth noting that some types of drugs used to treat IBD may have distinct effects on symptoms and microbial ecosystems. Because patients with IBD are usually advised to take cobalamin, Zhu et al. investigated the influence of cyanocobalamin (CNCBL) or methylcobalamin (MECBL) ingestion on DSS-induced IBD mice by 16S rRNA analysis in fecal samples. These authors found that a high concentration of CNCBL but not MECBL supplementation markedly aggravated IBD, with an increasing proportion of *Escherichia*/*Shigella* and a decreasing abundance of *Lactobacillus*, *Blautia*, and *Clostridium XVIII* [109]. This finding may provide a novel reference for treating IBD patients based on the microbiota in the clinic.

## 5. Conclusion

In summary, increasing gut microbiota-associated approaches in healthy and disease states may contribute to the discovery of ways to prevent or repair perturbations in host autoimmune diseases. Therapies targeting the gut microbiota may be effective in the future prevention or treatment of autoimmune diseases. In general, a progressive understanding of the dynamic interplay between the gut microbiota and the host will help in establishing highly individualized management for autoimmune disease patients and achieve better efficacy in clinical outcomes or even in discovering new therapeutic targets for RA. Dietary interventions, FMT, and live biotherapeutics targeting the corresponding gut microbiota will possibly become therapeutic treatments for autoimmune diseases. Metabolites derived from bacteria may also be used as potential therapies for both nonintestinal and intestinal autoimmune diseases, and correlations between the gut microbiota and its metabolic signatures may determine a predictive profile for autoimmune disease causation and progression.

Nonetheless, additional work is still needed, and whether changes in gut microbiota signatures are the cause or consequence of autoimmune diseases remains unclear. Furthermore, it is still uncertain which microbiota or metabolite alterations influence autoimmune diseases, and whether they can serve as biomarkers or therapeutic interventions requires further research. Although the current evidence supports that changes in the gut microbiota can affect the balance of Th17 cells and Tregs, which will influence pro-/anti-inflammatory cytokine levels [110], further work remains to be conducted to fully understand the mechanisms of the interaction between gut microbiota and the immune system. Moreover, different locations in the intestinal tract may differ in providing nutrient or physicochemical conditions for bacterial communities [111]. Thus, different microbial distributions along the gut may contribute to diverse changes in microbial communities in autoimmune diseases, but most studies have focused on fecal bacterial alterations. At present, the development of approaches to prevent autoimmune diseases by altering bacterial structure, including prebiotics, probiotics, and FMT, is still challenging. In addition, finding novel AhR agonists may be a logical next step to developing more effective probiotics to alleviate autoimmune diseases. Moreover, when using FMT therapies, it is crucial to explore whether there are influencing factors including but not limited to diet, sex, age, and geographical location. Administration of prebiotics or probiotics may have a beneficial effect on autoimmune diseases, but the mechanisms by which they interact with the host remain unresolved.

## Figures and Tables

**Table 1:** Autoimmune diseases and alteration of the gut microbiota composition.

Diseases	Species	Methods	Increasing microbiota species	Decreasing microbiota species	References
RA	Mouse	16S rRNA gene sequencing	*Desulfovibrio* *Mucispirillum* *Helicobacter* Lachnospiraceae *Rikenellaceae_RC9*	*S24-7* *Rikenella*	[60]
	Human	16S rRNA sequencing		Euryarchaeota	[54]
	Human	16S ribosomal DNA	*Collinsella*	Actinobacteria	[53]
	Human	Denaturing gradient gel electrophoresis	*Lactobacillus*		[57]
	Mouse	16S rRNA sequencing	Bacteroidaceae *Lachnospiraceae* S24-7		[47]

SLE	Mouse, human	16S rDNA sequencing	*Lactobacillus reuteri*		[62]
	Human	16S rRNA sequencing	Proteobacteria	Ruminococcaceae	[61]
	Human	16S ribosomal RNA gene sequencing	*Bacteroides*	Firmicutes/Bacteroidetes ratio	[112]
	Human	16S rRNA sequencing	*Streptococcus* *Lactobacillus* *S. anginosus* *L. mucosae* *Megasphaera*	*Faecalibacterium* *F. prausnitzii* *Cryptophyta* *Roseburia* *Bifidobacterium*	[66]
	Human	16S rRNA sequencing	*Ruminococcus gnavus*		[67]
	Human	Metagenomic analyses		Firmicutes/Bacteroidetes ratio	[69]
	Mouse	16S rDNA sequencing	Lachnospiraceae	*Lactobacillus* spp.	[47]

SpA	Human	16S rRNA gene- and ITS2-based DNA sequencing	*Escherichia*-*Shigella* *Veillonella* Lachnospiraceae NK4A136 group Dothideomycetes	*Prevotella* strain 9 *Megamonas* *Fusobacterium* Agaricales	[79]
	Human	16S ribosomal RNA amplicon sequencing	Dialister		[83]
	Human	16S ribosomal RNA gene sequencing	*Ruminococcus gnavus*		[84]

IBD	Human	Metagenomic sequencing	*Escherichia coli*	*Eubacterium rectale* *Faecalibacterium prausnitzii*	[101]
	Human	Tag-sequencing the 16S rRNA gene		*Roseburia* spp.	[93]
	Human	16S rRNA sequencing	Actinobacteria Proteobacteria (Enterobacteriaceae)	Firmicutes (Clostridiales)	[94]
	Human	16S rRNA sequencing	Proteobacteria	Firmicutes Actinobacteria	[95]
	Dog	16S rRNA gene next-generation sequencing	Paraprevotellaceae *Porphyromonas*		[91]
